# Perceptions of mental health nurses about psychosocial management of depression in adolescents, North West province, South Africa

**DOI:** 10.4102/hsag.v26i0.1528

**Published:** 2021-06-09

**Authors:** Precious C. Chukwuere, Leepile A. Sehularo, Mofatiki E. Manyedi

**Affiliations:** 1 Department of Nursing Science, Faculty of Health Sciences, North-West University, Mahikeng, South Africa

**Keywords:** adolescent, depression, management, mental healthcare, North West province, perception, psychosocial, South Africa

## Abstract

**Background:**

Depression in adolescents is a multifactorial global public health concern, with devastating consequences on the sufferer. The prevalence of depression amongst this age group is on the rise, and thus there is the need for greater attention.

**Aim:**

To explore and describe the perceptions of mental health nurses regarding the psychosocial management of depression in adolescents in North West province, South Africa.

**Setting:**

The study was conducted in two mental healthcare institutions and two mental healthcare units within two general hospitals in North West province, South Africa.

**Method:**

A qualitative, explorative, descriptive and contextual research design was used in conducting this study. Data were collected through focus group discussions from four groups of mental health nurses from each of the mental healthcare institutions and mental healthcare units with 18 mental health nurses. Data were analysed using Tesch’s open coding method.

**Results:**

Two themes emerged from the study: comprehensive psychosocial management and involvement of different stakeholders.

**Conclusion:**

The findings revealed clear psychosocial management for depression in adolescents. Adopting the findings of this study could improve depressive symptoms and curtail the prevalence of depression amongst adolescents in the North West province, South Africa.

## Introduction and background

Depression in adolescents is a multifactorial mental health problem that affects the life of the sufferer, families and the society, thus constituting a serious public health concern (Clow 2016:4). Developmental changes in adolescents (emotional, physical and cognitive changes) predispose them to high psychopathological conditions such as depression (Babore et al. 2016:982; Stikkelbroek et al. 2016:2). Depression is amongst the major causes of disability burden amongst adolescents (Collishaw et al. 2016:49). The World Health Organization (2017) maintains that depression is the third leading cause of disability amongst adolescents. An estimated 2.8% of children below the age of 13 years and 5.6% of adolescents aged 13–18 years are affected by depression (Hopkins et al. 2015:184). Researchers believe that adolescents with parents suffering from depression are three-to-four times at risk of suffering from the condition (Collishaw et al. 2016:50). Chukwuere, Pienaar and Sehularo (2020:125) state that stress related to academic activities is amongst the predisposing factors to depression in adolescents. Difficulties in coping with challenges resulting from breakup in relationships and stressful life experiences are also proximal factors to depression (Stikkelbroek et al. 2016:10).

Depression constitutes the most prevalent mental health problem amongst adolescents and can occur concurrently with anxiety (Nilsen, Eisemann & Kvernmo 2013:69). Ajaero, Nzeadibe and Igboeli (2018:73) state that depression amongst adolescents in South Africa has an annual prevalence rate of 20%, as reported by the South African Stress and Health (SASH) national representative. The Western Cape alone has an estimated 15% – 17% rate of depression amongst adolescents, while the North West province (NWP) has a prevalence rate of 7.45% amongst its rural population and 18.6% within the urban population. This calls for concerted efforts in providing lasting evidence-based psychosocial management measures to help in curtailing depression, encouraging resilience, help-seeking behaviours and reducing relapse.

As an emotional disorder, depression predisposes adolescents to social impairment, academic failures, substance abuse, heightened feeling of loneliness and reoccurrence at a later stage of life (Babore et al. 2016:3). Stikkelbroek et al. (2016:10–11) argue that depression leads to suicide ideations in adolescents. Suicide is the second leading cause of death amongst adolescents (Inman et al. 2019:68). According to Consoli et al. (2013:2), ‘an estimated 49% – 64% of adolescents commit suicide because of depression’. Early detection of depression amongst adolescents is a major headway to understanding the problem and propounding better management options (Collishaw et al. 2016:49; Dardas, Van de Water & Simmons 2018:555). Early detection facilitates the ability of the mental health nurses to evaluate the psychosocial risk factors around adolescents for adequate application of the best psychosocial management strategies (Hopkins et al. 2015:185).

The mental health nurses amongst other mental healthcare practitioners are faced with the challenges regarding the management of depression in adolescents (Knowles et al. 2015:9). Mitigation of depression in adolescents deserves more proactive measures from mental healthcare practitioners, such as mental health nurses, with regard to adopting comprehensive evidence-based (integration of best research evidence relying on scientific evidence guidelines for making decisions) psychosocial management frameworks for its management, considering the ravaging consequences in the society (Brownson et al. 2017:4; Straus et al. 2018:1).

Nassen et al. (2014:82) confirmed that psychosocial management measures are imperative for mental healthcare practitioners, such as mental health nurses, in mitigating depression amongst adolescents in NWP, South Africa. Nassen et al. (2014) posited that psychosocial management approaches are necessary for mental healthcare practitioners, especially mental health nurses, in managing depression in adolescents. Jones et al. (2018:2) further emphasised on the need of psychosocial management of depression in adolescents and affirmed that psychosocial management can potentially enhance adolescent resilience, thereby mitigating relapse. Psychosocial managements are social and psychological integrated programmes adopted by mental healthcare practitioners, such as mental health nurses, in the management of a wide range of mental health challenges, including depression in adolescents (Calear et al. 2016:467).

Psychosocial management is an appropriate line of management for depression in adolescents because of its multifactorial causes and accompanying devastating health challenges (Nilsen et al. 2013:70). Proper adoption of psychosocial management of depression in adolescents, such as psycho-education by mental healthcare practitioners, can enhance resilience of sufferers and families. Such adoption can also lead to an increase in management skills, strengthen communication, foster problem-solving mechanisms, increase crisis management and improve general health (Dardas et al. 2018:556). Despite the above explanations on psychosocial management, there is paucity of studies on psychosocial management of depression in adolescent from mental health nurses’ perspective in NWP, South Africa. This study focused on the exploration and description of perceptions of mental health nurses regarding the psychosocial management of depression in adolescents in NWP, South Africa, as it could facilitate the improvement of depressive symptoms amongst sufferers, curtail the prevalence of depression through provision of baseline information for mental healthcare practitioners, and stimulate further studies on this perspective.

## Research methodology

### Research design

A qualitative, explorative and descriptive contextual research design, as explained by Polit and Beck (2017:372), was used to explore and describe the perceptions of mental health nurses about the psychosocial management of depression in adolescents in NWP, South Africa. This research design enabled the researcher to generate quality data from participants. In addition, the research design enabled for the exploration and description of the perceptions of mental health nurses about psychosocial management of depression in adolescents in NWP, South Africa, and hence is best for the study.

### Context

This study was conducted in two mental healthcare institutions and two mental healthcare units in NWP. The province has four districts: Dr Kenneth Kaunda, Ngaka Modiri Molema, Bojanala and Dr Ruth Segomotsi Mompati districts. The study was conducted in the outpatient units of the two mental healthcare institutions, situated in Dr Kenneth Kaunda and Ngaka Modiri Molema districts. Data were also collected in the outpatient units of the two mental healthcare units attached to the general hospitals in Bojanala and Dr Ruth Segomotsi Mompati districts.

### Population and sampling

The study population included mental health nurses working in the mental healthcare institutions and mental healthcare units in NWP, South Africa. A non-probability purposive sampling technique was used to select the participants (Polit & Beck 2017:372). This sampling method was used in this study to select mental health nurses from two mental healthcare institutions and two mental healthcare units attached to two general hospitals in NWP. This sampling technique was considered appropriate for the study because it allowed the researcher to purposefully select participants based on their ability and positions to appropriately answer the research questions. In addition, purposive sampling enabled the researcher to recruit participants who were able to voice their opinions, perceptions and views about the study. Eighteen mental health nurses were recruited from the two mental healthcare institutions and two mental healthcare units attached to the two general hospitals in NWP, with the help of administrative officers (mediator). The nurses were selected because they were employees of the mental healthcare institutions and mental healthcare units and had worked for more than 5 years in these institutions and units. Both male and female nurses were considered for the study. Nurses were selected because they could understand and speak English or Setswana. The nurses were all registered with the South African Nursing Council (SANC) as mental health nurses (psychiatric nurses).

### Data collection

Data were collected from mental health nurses through focus group discussions. A focus group discussion is ‘a flexible method of data collection that brings participants with different views into contact, allowing them the room to share their perceptions regarding the research question’ (Ary et al. 2014:176). Focus group discussions were conducted with 18 mental health nurses from two mental health institutions and two mental healthcare units attached to general hospitals in NWP, South Africa. Three of the groups consisted of five mental health nurses each, while the other one consisted of three mental health nurses. The number of participants was smaller in the last group as there were a smaller number of mental health nurses in this mental healthcare unit. The focus group discussions were held in a private venue in each of the study areas. Group members were all assigned codes, which they used throughout the data collection process to ensure anonymity.

Each focus group discussion session lasted for 40 min to 1 h, with questions calmly asked, which enabled the participants to understand before responding while follow-up or probing questions were further asked for clarity and in-depth information. Official permission to use a tape recorder was sought and obtained from the participants during data collection. The researcher endeavoured to write field notes that helped in capturing all the information from the participants regarding the study. The principle of data saturation was adhered to in this study. Data saturation refers to the point at which participants or groups of participants at any level of data collection are unable to put forth new information pertaining to the research questions (Creswell 2014:296).

### Data analysis

Data were analysed separately by the researcher and an independent co-coder. The co-coder is an expert in qualitative data analysis. Tesch’s (1990) open coding method was used in accordance with Creswell (2014:246). Steps involved in transcribing and analysing the raw data obtained from the focus group discussions were as follows: transcribing the raw data, reading thorough in order to make sense of the data, reflecting on the meanings of the data, removing junks and preparing for analysis. Furthermore, coding the data; generating concepts, categories and themes; organising the concepts, categories and themes; and categorising and validating themes in order to ensure ideas from participants were properly represented. At the end of data analysis, the researcher and the co-coder reached a consensus over the themes and categories generated. The co-coder was involved after the data were transcribed word-by-word by the researcher.

### Ethical considerations

The study was approved by the Scientific Committee of the School of Nursing Science (SONS) and the Health Research Ethics Committee of the North-West University (HREC Reference number: NWU-00448-19-A1). Approval was obtained from the Department of Health. The management of the two mental healthcare institutions in Ngaka Modiri Molema and Dr Kenneth Kaunda and the mental healthcare units within the general hospitals in Dr Ruth Segomotsi Mompati and Bojanala districts approved the study. Mental health nurses signed voluntary consent forms before participating in the study. All participants adhered to the group rules as follows: everyone had the right to express themselves constructively regarding the topic of discussion; no need to raise issues not related to the topic of discussion; no separate conversation or abusive language against participants; no interruption during discussions; group members requested to respond to questions one after the other to avoid disrupting the views and thoughts of others; all discussions within the group to remain within the group (non-disclosure) and all team members given the right to ask questions for clarity.

### Trustworthiness

Trustworthiness is about ascertaining the credibility, dependability, confirmability, transferability and authenticity of a study (Ary et al. 2014:442). Credibility was ensured through member checking, removing errors and ensuring clarity of the findings. A relationship based on trust was maintained during data collection, enabling participants to comfortably respond to the questions. Dependability was ensured through careful conceptualisation of the study, data collection, analysis, interpretation and reporting of the findings. Confirmability was ensured through careful recording of focus group discussions, transcription of the raw data verbatim, careful coding and interpretation of data (Creswell 2014:251). Transferability was ensured through detailed explanation of the context, target population, sampling technique, data collection and analysis methods. Authenticity was ensured through attentive listening to mental health nurses and using probing questions to obtain in-depth information.

## Results and discussion

### Demographic characteristics of participants

Eighteen mental health nurses (14 women and 4 men) participated in the four focus group discussions. All the mental health nurses who took part in the study had been working within the different facilities for more than 5 years and were registered with the SANC as mental health nurses, and thus met the inclusion criteria of the study.

The themes and categories that emerged from the data are presented in Table 1.

**TABLE 1:** Themes and categories.

Themes	Categories
Comprehensive psychosocial management	School health education is a vital aspect of the management of depression Counselling services are vital for adolescents Effective problem-solving approaches can assist in the management of depression in adolescents Role-play for depression and its symptoms Services rendered should be adolescent-friendly Integrating mental health into primary healthcare
Involvement of different stakeholders	Peer education makes it more acceptable to adolescents Family support system enhances speedy recovery Role modelling by parents moulds adolescent behaviour Involvement of educators to facilitate recovery Involving community members Engaging media houses Collaboration with NGOs in improving mental health

NGO, non-governmental organisation.

#### Theme 1: Comprehensive psychosocial management

This theme emerged from responses obtained from mental health nurses with regard to the research question. The theme constitutes one of the perceptions of mental health nurses regarding the psychosocial management of depression in adolescents. The different categories are described below.

**School health education is a vital aspect in the management of depression:** During the focus group discussions, mental health nurses indicated that school health education is necessary for mitigating depression in adolescents. Schools play an important role in nurturing adolescents to better fit in society, including their emotional life control, through the different activities embedded in the school curricula. Participants maintained that ‘school health education is very important in mitigating the prevalence of adolescent depression; nurses must go to the schools, offer them education in the schools’ (Focus group 1, participant 5, female). They also maintained ‘schools should be teaching these children how to control their emotions in order to overcome depression’ (Focus group 3, participant 2, female).

Schools are helpful in curtailing depression in adolescents in society. To achieve this objective, school management teams should assist in developing strategies that can best help depressed adolescents in their recovery (Thomson 2018:39). Furthermore, Rentala et al. (2019:1082) argued that adequate school health education programmes improve depressive symptoms and other mental health related conditions in adolescents.

**Counselling services are vital for adolescents:** Counselling was perceived by mental health nurses as a strategy to boost coping mechanisms in adolescents and, therefore, effective in managing depression. Mental health nurses maintained the following: ‘Psychosocial management must be counselling because it is necessary, counselling must be involved with adolescents and also parents or teachers depending on the circumstances or the situation’ (Focus group 1, participant 1, male); ‘Using counselling is very important in the life of the adolescent; it will also help the child to get better’ (Focus group 4, participant 1, female):

‘[*W*]hoever that is doing the counselling, must teach them in that manner that is understandable like maybe, not being harsh, show empathy, like being in their shoes, like treat them well.’ (Focus group 1, participant 4, male)

According to Wilkinson, Cestaro and Pinchen (2018:134), counselling of adolescents is interpersonal and a time-limited psychological therapy focusing mainly on two links between depressive symptoms and interpersonal relationship. Enhancing one’s understanding of these links makes possible improving depressive symptoms in adolescents. Hussin, Mahmud and Karim (2020:53) maintained that counselling could be helpful in emotional management of adolescents suffering from depression. The counselling could be held in groups or on individual basis. Glasheen, Shochet and Campbell (2016:108) further argued that ‘counselling sections are effective in ameliorating depressive symptoms among adolescent’. The counselling sessions can also be carried out through online platforms, which is considered as effective as individual face-to-face counselling.

**Effective problem-solving approaches can assist in the management of depression in adolescents:** Participants indicated that there is a need for adolescents to use effective problem-solving approaches for the psychosocial management of their condition. Depression in adolescents is a mental health condition, which affects the emotion of the sufferer (Rice et al. 2019:175). Thus, proper management of depression in adolescents necessitates effective problem-solving approaches to assist the sufferer in the recovery process. These problem-solving approaches should be geared towards addressing problems of sufferers as they emanate. Effective problem-solving approaches can be channelled towards addressing issues that aggravate symptoms in sufferers as captured in the following excerpts:

‘[*W*]here this trending of a thing must be about the adolescent depression, eeeem what causes it and they must come up with their problem, they must come up now with their problems so we are able to manage it for their recovery.’ (Focus group 1, participant 1, male)

‘[*T*]each them everything about depression, then they will be able to deal with it because they will be knowing the signs and symptoms and the cause, then they will be able to know what to avoid and what not to avoid, to know what to do and what not to do.’ (Focus group 2, participant 1, male)

Adopting effective problem-solving approaches in addressing depression in adolescents can have a positive influence in mitigating depressive symptoms (Dunne et al. 2019:113). Sahin and Adana (2016:1273) further maintained that ‘an effective problem-solving approach enables identification of the level of depression of adolescents, thus, assisting in the administration of the right management strategy’.

**Role-play for depression and its symptoms:** Participants indicated that mitigating depression in adolescents in society can be multi-factorial because of the numerous factors that predispose adolescents to depression. These factors necessitate the adoption of best media for the dissemination of appropriate management measures to enable the adolescents lead a better life. Hence, for sufferers to properly comprehend their depressive state, appropriate teaching can be conveyed through role-play. Mental health nurses maintained as follows:

‘[*U*]sually, young people, if you want to get involved in their depression management, they like role-play so when you come to the clinics, usually, they play games, let’s say maybe now you want to talk about adolescent depression and managements.’ (Focus group 1, participant 2, female)

Mental health nurses should be able to effectively convey the needed information regarding depression in adolescents and its management, thus assisting in alleviating symptoms on the sufferer. Boyd et al. (2018:351) stated that depression in adolescents is a prevalent mental health disorder that warrants mental healthcare providers to be at the front line of management. The condition can thus be properly managed by engaging adolescents to promptly identify their depressive symptoms.

**Services rendered should be adolescent-friendly:** Mental health nurses emphasised the need for adolescent-friendly services to foster adolescent consultation geared towards managing depression as captured in the following excerpts:

‘We also need friendly user services in our facilities.’ (Focus group 1, participant 3, female)

‘When sufferers come to our facilities, they should be user-friendly, our services should accommodate the adolescents.’ (Focus group 1, participant 2, female)

‘Mental health care practitioners should be friendly with the children, give them attention, and show them that you are with them.’ (Focus group 4, participant 2, female)

The South African healthcare system is inequitable and polarised, post-apartheid, making it difficult for users, including adolescents, who are depressed, to get user-friendly services (Van Rensburg 2014:1). According to Baltag and Sawyer (2017:309), adolescents encounter many barriers in accessing healthcare services, especially services relating to mental healthcare conditions; however, these barriers can be improved by standard-driven or indicator approaches. Standard-driven or indicator approaches are capable of enhancing the quality of healthcare services received by adolescents. Through quality standards and indicators, more services become transparent and healthcare service providers become more accountable. Ambresin et al. (2013:670) reported that healthcare institutions in the United States of America are already embracing adolescent user-friendly services, including mental healthcare facilities.

**Integrating mental health into primary healthcare:** The study participants emphasised the need to integrate mental health into primary healthcare. Psychosocial management of depression in adolescents cuts across different strategies channelled towards curtailing depression. During focus group discussions, mental health nurses indicated the following:

‘And I have to say to them that they must include mental health in tertiary health care institutions to primary health care units.’ (Focus group 1, participant 1, male)

‘[*I*]n order to improve adolescent mental health conditions, especially depression, those nurses who have done advanced psychiatric nursing, must be enough so that they can be distributed to the primary health settings, and must be the ones that will be administering psychosocial managements to the adolescents and families.’ (Focus group 1, participant 1, male)

‘[*I*] think primary health care units must be involved in disseminating psychosocial management for adolescent depression and other mental health problems affecting adolescents because of that, adolescent mental health psychosocial management must be integrated into primary health system and strictly be adhered to.’ (Focus group 4, participant 3, female)

Primary healthcare services are closer to the masses, and hence are better placed to render psychosocial management of depression in adolescents. Primary healthcare facilities are expected to be diligent in the discharge of their duties, such as creating awareness regarding depression in adolescents and other common public health concerns (World Health Organization 2018:18). Primary healthcare facilities considered appropriate for the integration of quality improvement programmes geared towards the management of mental health conditions amongst adolescents, including depression, are effective in providing communities with accessible psychosocial management (Lam et al. 2016:325).

#### Theme 2: Involvement of different stakeholders

Involvement of different stakeholders is one of the strategies in the psychosocial management of depression in adolescents. The question sought to understand the views of mental health nurses with regard to the psychosocial management of depression in adolescents. This theme provided the following categories: adolescents are more comfortable with peer education, family support systems enhance speedy recovery, role modelling by parents moulds adolescent behaviour, involvement of educators to facilitate recovery, involving community members, engaging media houses and collaboration with non-governmental organisation (NGOs) in improving mental health.

**Adolescents are more comfortable with peer education:** The focus group discussions revealed the need for peer education in effectively managing depression in adolescents. Peers play an important role in influencing their fellows, which is underpinned in many reasons, such as their ability to communicate in the most understandable language amongst themselves, amongst others. Mental health nurses ascertained that ‘they can teach the other ones about depression’ (Focus group 2, participant 5, female). Another mental health nurse added the following:

‘[*G*]enerally speaking, adolescents learn from themselves most of the time, they teach each other, so even though most of the things they teach each other are odd things, they can also teach theirs about the condition that are more common in the society such as depression.’ (Focus group 2, participant 5, female)

‘[*A*]nd that reminds me, we can also look at encouraging peer-to-peer talk about this depression, if we can educate peers that they may go out and educate orders, I think it will help in managing depression in adolescents. Like this, peer groups understand themselves better.’ (Focus group 4, participant 2, female)

According to Markova and Nikitskaya (2017:36), it is imperative for adolescents to understand the influence of peers in strengthening each other’s resolve in coping during emotional difficulties, especially during depression. Adequate understanding of such roles will enable them to watch each other’s back during depressive state, which, in turn, enhances recovery. The study revealed that peer-to-peer programmes for managing depression are effective because adolescents feel comfortable talking to each other about their struggles with depression. Peer leaders in such depression management programmes serve as a positive role model to those suffering from depression, and help to shape their behaviours and social norms that are negatively affecting their recovery (Parikh et al. 2018:491).

**Family support systems enhance speedy recovery:** Participants indicated that adolescents need support systems to manage depression. Support systems could be understood to represent psychosocial management as they represent the necessary support needed by the adolescents with depression for recovery as captured in the following excerpts by mental health nurses:

‘[*P*]sychosocial management of depression in adolescents entails the use of the adolescent’s immediate environment such as the family environment and the friends in caring for the depressed adolescent in order to foster progressive recovery.’ (Focus group 1, participant 4, female)

and:

‘To properly manage depression, I think at home, parents should also be part of the management programme.’ (Focus group 3, participant 1, female)

Other mental health nurses maintained as follows:

‘[*S*]o, the families, for them, they can help out, let’s be open to our kids, let’s give them the platform, we can come up with reasons, let’s involve in terms of running of the whole family.’ (Focus group 2, participant 1, male)

and:

‘[*I*] think psychosocial management of adolescents should entail involvement of all the people that are around the patient in the family, how they care for the child, how they show understanding of what the child is passing through.’ (Focus group 4, participant 2, female)

Adolescents benefit from family support during periods of mental challenges, such as depression, through modifying depression-associated risk factors, such as family stress, thus assisting the individual in leading a better life (Carr 2016:467). Corcoran (2017:14) also ascertained that family support is crucial in managing depression amongst adolescents. Adequate support from members of the family ensures a sense of belonging in the life of an adolescent suffering from depression. Depression can be caused by various factors, which could have emanated from the environment or elsewhere. Hence, adequate family support is imperative. Furthermore, Cregeen (2018:240) added that besides family support, in managing depression, cognitive behavioural therapy (CBT) and interpersonal therapy are also important.

**Role modelling by parents moulds adolescent behaviour:** Mental health nurses emphasised the importance of role modelling in the lives of adolescents. Role modelling can help in channelling their energy appropriately to prevent snapping into depression or mitigating symptoms of depression, thus leading to recovery as captured in the following excerpt:

‘[*W*]e, as health care professionals and also as parents, we have to be their role models, what is the use for me to be at home and me and my husband are drinking liquor in front of the children? If they start drinking, what is it that I can tell them, that they must stop, how can I say to them that they must not drink because they copied from me.’ (Focus group 1, participant 4, female)

According to Johnson et al. (2016:136–137), adolescents tend to learn positive things from their role models, and integrate them into their lives for adequate coping in adverse situations, such as when they suffer from depression. The ability to internalise emotion-regulation strategies against depression can be enhanced by things adolescents learn from parents (Morris et al. 2017:235). Thus, parental role modelling attributes and parenting styles enhance emotional stability of adolescents, provide favourable emotional regulation environment and foster recovery from depression.

**Involvement of educators to facilitate recovery:** Participants indicated that adolescents spend most of their time in school, and thus need to involve educators in the management of their mental conditions such as depression. The psychosocial management of depression amongst adolescents requires the collective effort of everyone around their recovery, as captured in the following excerpts by mental health nurses:

‘It is very important to involve teachers in the psychosocial management of depression in adolescents.’ (Focus group 3, participant 3, female)

‘Psychosocial management should involve teachers at schools in order to reduce symptoms and enhance recovery.’ (Focus group 1, participant 1, male)

‘[*T*]he teacher spends quality time with adolescents in school, they watch over their general well-being, therefore, they can help in educating them on depression and its management. With that, those suffering from depression can have their symptoms improved.’ (Focus group 4, participant 3, female)

Teachers can also be equipped in administering management to adolescents in schools for depression because they spend a good number of hours with students in the school daily, and hence teachers can account for their emotional behaviour (Yu et al. 2016:115). Abraham and Scaria (2018:694) advocated equipping teachers with critical skills to manage depression in schools, including depression management programmes in the school curriculum. Adding such management programmes in the school curriculum is necessary because teachers play a crucial role in nurturing adolescents emotionally.

**Involving community members:** Participants emphasised the need for the whole community to be involved in managing depression in adolescents. Mental health nurses who participated in the focus group discussions indicated the following:

‘[*W*]e have community psychiatric nurses who help nurses in the community; I think they should visit schools, churches and teach people about the conditions affecting adolescents such as depression and help them on how to deal with symptoms.’ (Focus group 2, participant 3, female)

‘[*I*]n the community, also we have community leaders, I think if they are being educated on adolescent depression and other mental health conditions affecting adolescents and the necessary management, that will help to improve their mental health.’ (Focus group 3, participant 3, female)

There is a need to engage communities on adolescent depression education and other mental health challenges by educating them on every necessary information that can foster adequate psychosocial management of depression and its mitigation (Theron, Theron & Malindi 2012:64). Lam et al. (2016:325) stated that community engagement on depression in adolescents and other mental health conditions is effective in improving the symptoms of sufferers, including stress on families and mitigating associated stigmas.

**Engaging media houses:** Most participants indicated for the need to engage media houses for the psychosocial management of depression in adolescents. This is an aspect of psychosocial management that can assist adolescents with depression to better understand their condition and lead a better life as captured in the following excerpts by mental health nurses:

‘[*A*]s things are so modernised nowadays, I think if we go to radio stations, most adolescents once you go there, talk about this adolescent depression and all the causes of depression among adolescents, most of them, they do call and speak to you over the radio.’ (Focus group 1, participant 1, male)

‘Also, I think those social media must play a big role here.’ (Focus group 2, participant 1, male)

‘Like now, we have loads of radio stations but how many times do we go to the radio station so that young people can call?’ (Focus group 1, participant 2, female)

Qassim, Boura and Al-Hariri (2018:402) argued that media houses present fast and wide avenues for circulation of information that are highly influential and capable of assisting adolescents suffering from depression in their recovery. These influences of media houses, through the circulation of mental health promoting information, are believed to emanate from public belief on media houses.

**Collaboration with non-governmental organisations in improving mental health:** Participants indicated that the psychosocial management of depression in adolescents requires collaborative activities of different bodies, such as NGOs, as captured in the following excerpts: ‘Like at the clinic, we need these NGOs that are working with the department like LOVE LIFE’ (Focus group 1, participant 2, female); ‘You have to make sure that they visit adolescents at schools or at home so that they can assist them with their needs, this will reduce their depressive symptoms’ (Focus group 1, participant 3, female).

Leung (2019:120) posited that the psychosocial management of depression in adolescents can be fostered through collaborative efforts of NGOs, government and celebrities in circulating health-improving information that will benefit sufferers. Collaborative efforts of organisations in the management of depression help in providing vital information on mental health, including information on the most reliable management measures (Richardson et al. 2014:8). Adequate collaboration of NGOs with other sectors, such as education, welfare, justice and labour, is crucial in the implementation of global mental health and development policies, and to foster the availability of information on the management of depression (Collins et al. 2013:1).

### Limitations of the study

The findings of this study cannot be generalised; rather similar studies should be conducted in different contexts in other provinces of South Africa. However, other provinces can use the findings of this study to manage depression in adolescents. Some mental health nurses refused to participate in the study for different reasons. For ethical reasons, the researcher respected their decisions not to participate in the study.

## Conclusion

The aim of this study was to explore and describe perceptions of mental health nurses on psychosocial management of depression in adolescents in NWP, South Africa. Two themes (comprehensive psychosocial management and involvement of different stakeholders) emerged from the study and were discussed with different categories, thus providing a comprehensive understanding of the study. The findings revealed the need for psychosocial management of depression to encompass school health education, counselling, problem-solving, adolescent-friendly user services in facilities, peer education, using immediate environment of adolescents to foster recovery and role modelling. The study further provides nurses’ perceptions of psychosocial management of depression in adolescents, which is necessary to mitigate the prevalence of depression in society. Despite studies on depression in adolescents gaining significant grounds, such as in the area of the prevalence of depression in adolescents, this study focused on the psychosocial management of depression amongst young people in society. The findings of this study could stimulate further research on depression in adolescents, such as in exploring the mental health nurses’ willingness to adopt psychosocial management in their management of depression in adolescents. Hence, it is recommended for the mental health nurses to prioritise the adoption of psychosocial management in their management of depression in adolescents to foster the adolescents’ development of appropriate coping skills in the province against depression.
